# ‘*Candidatus* Liberibacter asiaticus’ Accumulates inside Endoplasmic Reticulum Associated Vacuoles in the Gut Cells of *Diaphorina citri*

**DOI:** 10.1038/s41598-017-16095-w

**Published:** 2017-12-05

**Authors:** Murad Ghanim, Diann Achor, Saptarshi Ghosh, Svetlana Kontsedalov, Galina Lebedev, Amit Levy

**Affiliations:** 1 Department of Entomology, Volcani Center, Rishon LeZion, Israel; 2 Citrus Research and Education Center, University of Florida, Lake Alfred, FL USA; 3 Department of Plant Pathology, University of Florida, Gainesville, FL USA

## Abstract

Citrus greening disease known also as Huanglongbing (HLB) caused by the phloem-limited bacterium ‘*Candidatus* Liberibacter asiaticus’ (CLas) has resulted in tremendous losses and the death of millions of trees worldwide. CLas is transmitted by the Asian citrus psyllid *Diaphorina citri*. The closely-related bacteria ‘*Candidatus* Liberibacter solanacearum’ (CLso), associated with vegetative disorders in carrots, is transmitted by the carrot psyllid *Bactericera trigonica*. A promising approach to prevent the transmission of these pathogens is to interfere with the vector-pathogen interactions, but our understanding of these processes is limited. It was recently reported that CLas induced changes in the nuclear architecture, and activated programmed cell death, in *D*. *citri* midgut cells. Here, we used electron and fluorescent microscopy and show that CLas induces the formation of endoplasmic reticulum (ER)-associated bodies. The bacterium recruits those ER structures into Liberibacter containing vacuoles (LCVs), in which bacterial cells seem to propagate. ER- associated LCV formation was unique to CLas, as we could not detect these bodies in *B*. *trigonica* infected with CLso. ER recruitment is hypothesized to generate a safe replicative body to escape cellular immune responses in the insect gut. Understanding the molecular interactions that undelay these responses will open new opportunities for controlling CLas.

## Introduction

*Candidatus* Liberibacter bacterial species are phloem-limited, Gram-negative, unculturable bacteria vectored by psyllids^1–3^. These bacterial species have been associated with serious diseases of citrus, tomatoes, potatoes and other solanaceous crops. ‘*Candidatus* Liberibacter asiaticus’ (CLas)^4,5^ is implicated in causing the most serious disease of citrus, citrus greening disease, also referred to as Huanglongbing (HLB). This bacterium is vectored by the Asian Citrus Psyllid, *Diaphorina citri* Kuwayama^4^. Worldwide, *D*. *citri* and HLB have spread to most citrus growing regions. In the US, this disease threatens the future of Florida’s citrus industry^4^, and the pathogen and vector are spreading to new areas. ‘*Candidatus* Liberibacter solanacearum’ (CLso), is another closely related bacterium causing diseases in solanaceous and umbelliferous crops. Zebra chip, caused by CLso is an emerging disease which has caused significant economic losses, by reducing both yield and quality of potato crops^6^. Haplotypes of CLso infecting solanaceous crops are transmitted by the potato/tomato psyllid *Bactericera cockerelli* in North America and New Zealand, whereas haplotypes infecting umbelliferous crops like carrots, fennel and celery^7^, are transmitted by *Trioza apicalis* in Northern Europe and *Bactericera trigonica* in the Mediterranean and Middle East. Current management options for the diseases caused by CLso and CLas are limited and heavily rely on the application of chemical insecticides for controlling psyllid populations. However, those strategies are ineffective for the most part because of application problems, development of insecticide resistance among psyllid populations and the great threat to the environment and to beneficial organisms^4^. Developing efficient knowledge-based strategies to disrupt CLas and CLso transmission by their psyllid vectors represent an improved strategy to control the disease without relying on chemical sprays.

CLas and CLso are transmitted by psyllids in a persistent propagative manner. Several parameters for CLas interaction with *D*. *citri* including acquisition, retention, latent period and transmission were determined^8^. These results indicated that *D*. *citri* which acquire CLas as adults are poor vectors of the pathogen compared with adults that acquired the pathogen as nymphs, suggesting that bacterial multiplication during the nymphal stages is essential for efficient transmission^9,10^. CLas was detected in various *D*. *citri* organs, including the salivary glands, hemolymph, filter chamber, midgut, fat and muscle tissues, and ovaries^11^, suggesting propagation of the bacterium within insect tissues^12,13^. Parameters for CLso transmission by *B*. *cockerelli* were also investigated, but with fewer details than CLas. A recent study has shown that CLso invades the digestive and salivary systems of nymphs and adults of *B*. *cockerelli*, and that the bacterium employs endo/exocytosis-like mechanisms for circulation and transmission within the insect^14^. Transmission rates of the CLas and CLso probably depend on the ability of the bacteria to multiply within insect tissue and reach sufficient titers for transmission and on the ability to cross barriers during the transmission pathway especially the gut-hemolymph and the hemolymph-salivary glands barriers^12–14^. In addition, an important factor that determines the transmission efficiency of pathogens including CLas and CLso by their vectors is the response that the bacterium would trigger in the psyllid when it is acquired and retained, especially defense and immune responses. It was recently reported that CLas induced programmed cell death (apoptosis) and some necrosis in the insect gut^13^. Bacteria in general are known to induce many stress responses and activate molecular pathways in their hosts upon infection^15^. Some of these responses, like apoptosis, are immune responses targeted to destroy or limit the bacterial infections and spread in the insect. Several upstream events that lead to apoptotic or necrotic responses start in the Endoplasmic reticulum (ER). ER has several functions in the cell, most importantly the synthesis of transmembrane proteins, their correct folding and secretion. Under stress conditions, unfolded proteins accumulate and lead to the unfolded protein response (UPR), a cascade of events activated with the aim for restoring cell homeostasis^16,17^. In the case that homeostasis is not restored, subsequent signaling pathways that start in the ER are activated, leading to apoptosis^16^. One of the known stresses that induce UPR in the ER is the infection with viruses and bacteria, and it has been shown that bacteria and viruses can interact with the ER and employ this organelle for their own replication. *Legionella pneumophila* and *Brucella* spp. are the best examples of bacteria that were shown to occupy ribosome-studded intracellular vacuoles, confirmed to be derived from the ER. By intercepting vacuoles trafficking between the ER and the Golgi apparatus, these bacteria-containing vacuoles were shown to fuse with the ER-derived vacuoles to form vacuoles that have features of secretory compartments^18^. However, the roles played by the host ER during plant pathogen transmission were not explored.

In this work, we used confocal and electron microscopy, and show that CLas exploits the ER in *D*. *citri* to form vacuoles in the psyllid gut cells, and that these vacuoles develop and grow, possibly for CLas persistence and replication along the transmission pathway. We could not detect similar responses with CLso in *B*. *trigonica*. We suggest that ER is recruited by CLas in order to generate a safe replicative niche.

## Materials and Methods

### Insects, plants, CLas and CLso materials

*D*. *citri* populations used in this study were obtained from Dr. Bill Dawson (University of Florida). The populations were maintained under controlled conditions on *Citrus macrophylla* as described in^19^. Presence of CLas in the individual infected psyllids in the population was confirmed by qPCR using an ABI 7500 (Applied Biosystems) real-time PCR instrument, as described in ref.^19^.

CLso (haplotype D) free and infected *B*. *trigonica* populations used in this study were collected from carrot fields in southern Israel during the summer of 2015. The insects were transferred to celery plants, a suitable host for both *B*. *trigonica* and CLso that were maintained under controlled conditions inside insect-proof cages inside environmentally controlled growing chambers with a twelve-hour photoperiod. The presence of CLso in the infected insect populations, and the infection of plants, were verified by extracting total DNA from single *B*. *trigonica* insects or plant leaflets using 500 µl of CTAB buffer (2% cetyl trimethylammonium bromide, 1% polyvinyl pyrrolidone, 100 mM Tris-HCl, 1.4 M NaCl, 20 mM EDTA). qPCR was performed using CLso -specific primers (F- 5′-CACCCAACAGCTAGCACTCA-3′, R- 5′-TCAACCTTGGAACTGCCTTT-3) by amplifying 221 bp of the 16 s rDNA gene. Thermal cycling conditions of 95 °C for 20 seconds, 58 °C for 20 seconds, 72 °C for 25 seconds–for 40 cycles were used.

### Transmission electron microscopy

*D*. *citri* and *B*. *trigonica* abdomens were cut from whole adults reared on infected orange seedlings with CLas or infected celery with CLso, respectively, at their 1-week age under a stereomicroscope. The dissected abdomens were fixed for 16 h in 1X PBS buffer containing 4% (v/v) paraformaldehyde and 0.1% (v/v) glutaraldehyde; dehydrated; and embedded^20^ in Agar100 resin (Agar Scientific). 90 nm ultrathin sections mounted on copper grids were stained with 2% (w/v) uranyl acetate and lead citrate^21^ and examined using the FEI Morgagni 268, Transmission Electron Microscope (FEI, Hillsboro, Oregon).

### Immunogold labelling

Adult psyllids from HLB+ citrus plants were collected and anesthetized with acetone. Wings, legs and tip of abdomen were removed before placing them in Karnovsky’s fixative overnight at 4 °C. They were then dehydrated in ethanol, infiltrated with LR White resin over three days, then embedded and cured in a 70 °C oven. One micrometer sections were made with glass knives and stained with methylene blue/azure A followed by basic fuchsin. These sections were examined for location of gut and examined using an Olympus BX61 compound microscope (Cambridge Scientific Products, Watertown, MA) and photographed using an OMAX CMOS 14 mp digital camera. For TEM, ultrathin sections of midgut were prepared with a diamond knife on the same ultramicrotome, stained with 2% aqueous uranyl acetate, post-stained with Reynolds lead citrate, viewed and photographed on a Morgagni 268 transmission electron microscope. To confirm the identity of the bacteria, gold labeling was performed using a polyclonal antibody to an outer membrane peptide of Liberibacter (anti ompA pab; Abnova). Grids were placed on blocking buffer consisting of phosphate buffer saline, 0.1% triton X and 1% bovine serum albumin (PBST-BSA) for 15 min, drained on filter paper then incubated for 1 hr on 1:250 polyclonal primary antibody:PBST-BSA and rinsed 3X on drops of PBST-BSA 5 min each drop. For secondary antibody, grids were incubated 30 min on goat anti-rabbit 10 nm gold in PBST-BSA 1:10 drops, rinsed three times with PBST-BSA drops, 5 min each, rinsed two times with distilled, filtered water drops, 5 min each, then stained with 2% uranyl acetate and post stained with Reynolds lead citrate^21^.

### Psyllid gut dissections and microscopy

Staining of psyllid midgut cells using a variety of cell biology probes was performed to localize both CLas and CLso relative to other cell compartments, and to assess the impact of both bacteria on adult *D*. *citri* midgut cells and other tissues. Guts were dissected from adults in 1x phosphate buffered saline (1xPBS; pH 7.2) under a dissecting stereomicroscope by using depressed glass wells and fine entomological needles. A sufficient number of midguts for each analysis (20 or more) were washed 2–3 times with 1x PBS for further processing.

Nuclei in the midgut were stained with 4′,6-diamidino-2-phenylindole (DAPI) solution at 0.1 mg/ml in 1x PBS (pH 7.2). The guts were then transferred to slides, mounted whole in 1xPBS and viewed using a Leica SP8 laser-scanning confocal microscope (Leica Microsystems Inc., Buffalo Grove, IL, USA). Optical confocal sections (100 μm thick) were acquired from a randomly selected subset of the gut specimens for better visualization of the signal. Both CLas and CLso CLSM images following immunostaining (See below) were collected using the same Leica SP8 laser-scanning confocal microscope. Samples were imaged using lasers with excitation wavelengths for their respective fluorescent reporting range: Green fluorescence was excited with a 488-nm argon laser, and emission was detected at 500 to 530 nm. Red fluorescence was excited with a 561-nm diode-pumped solid-state (DPSS) laser, and emission was detected at 590 to 630 nm. Blue fluorescent protein was excited with near UV diode 405-nm laser, and emission was detected at 475–501 nm. Leica Application Suite - LAS X (Leica Microsystems Inc., Buffalo Grove, IL, USA) was used to collect z-stacks composed of optical sections at a 1024 × 1024 resolution. Images were exported as TIFF files to produce photographs for publication using Adobe Photoshop software.

### Immunolocalization of CLas and CLso and ER staining

Immunolocalization and ER staining were used to visualize CLas and CLso in CLas- and CLso exposed midguts, relative to the ER inside midgut cells. Healthy and CLas- or CLso-exposed *D*. *citri* or *B*. *trigonica* midguts, respectively, were dissected on glass microscope slides under a dissecting microscope in 1x PBS and fixed in 4% paraformaldehyde for 30 min at room temperature. Midguts were permeabilized by adding 0.1% Triton X-100 for 30 min at room temperature. The midguts were washed three times with PBST (1x PBS with 0.05% Tween 20), blocked for 1 h at room temperature with blocking buffer (PBST with 1% [w/v] bovine serum albumin), and incubated overnight at 4 °C with anti-CLas outermembrane protein (OMP) polyclonal primary antibody raised in rabbit (Abnova, PAB15989), which also detected CLso in *B*. *trigonica* midgut cells. Midguts were then washed three times with PBST, incubated with goat anti-rabbit secondary antibody conjugated to Cy3 (Jackson Laboratories) for 1 h at room temperature, and washed again three times with PBST. The midguts were fixed again with the fixation solution used above, washed three times with PBST and incubated with 1 µM ER tracker (BODIPY FL glibenclamide, Molecular Probes, Thermo Fisher Scientific) staining solution for 15 min. The midguts were then washed three times with PBST and mounted in 1x PBS containing 0.1 mg/ml DAPI, covered with a coverslip, sealed with nail polish, and viewed using confocal microscopy as described above.

## Results

### Nuclei ultrastructure following infection with CLso

**Figure 1:**
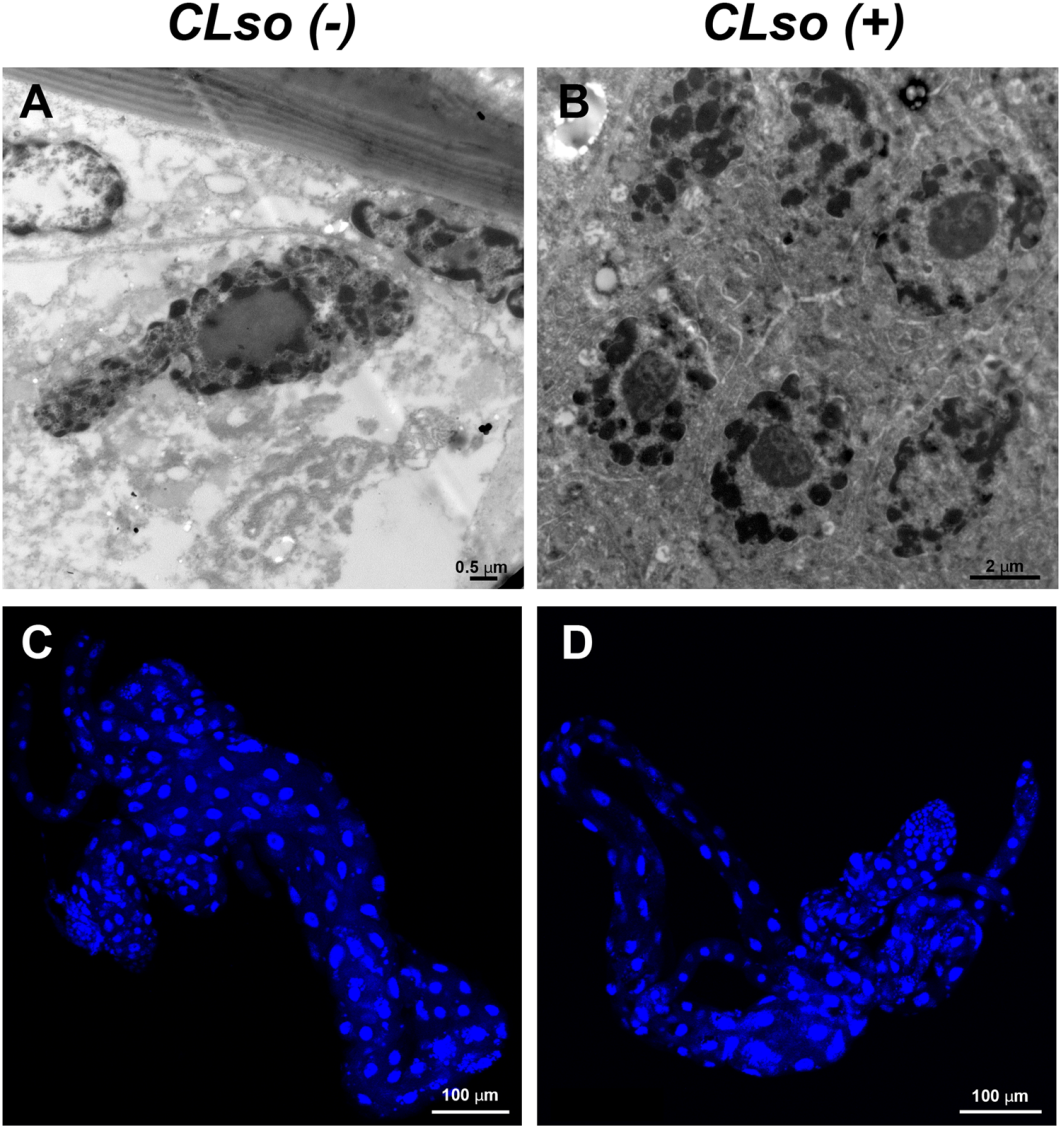
Normal structure of the nuclei in guts from CLso-uninfected (**A**,**C**) and CLso-infected (**B**,**D**) *B*. *trigonica* adults, as seen in TEM (**A**,**B**) and CLSM after nuclei staining with DAPI (**C**,**D**).

In a recent study, it was shown that CLas induced apoptosis in midgut cells of *D*. *citri*, leading to nuclear defragmentation^13^. In the current study, we used confocal microscopy and TEM analysis to examine the nature of these nuclear phenotypes in guts of *B*. *trigonica* that were reared on CLso infected (CLso+) or uninfected (CLso−) plants. We did not detect any cell death phenotype in the *B*. *trigonica* that were infected with CLso (Fig. 1). In TEM sections, nuclei in both CLso infected and uninfected *B*. *trigonica* midguts looked intact, and the chromatin appeared condensed with less or no fragmentation and dispersion (Fig. 1A,B). When stained with DAPI, gut nuclei appeared mostly normal with very little fragmentation in both infected and uninfected guts (Fig. 1C,D). Nuclei from *D*. *citri* that fed on healthy plants also looked normal in their shape and chromatin content, but some nuclei from infected *D*. *citri* were abnormal in their shape and structure, and the chromatin looked fragmented and sometimes dispersed around the fragmented nuclei, reminiscent of the phenotypes previously observed^13^ (Supplementary Fig. S1). Our results suggest that CLso does not induce in the psyllid gut cells the same nuclear phenotypes that were previously observed with CLas.

### CLas associates with and accumulates in ER-derived Liberibacter-containing vacuoles (LCVs)

**Figure 2:**
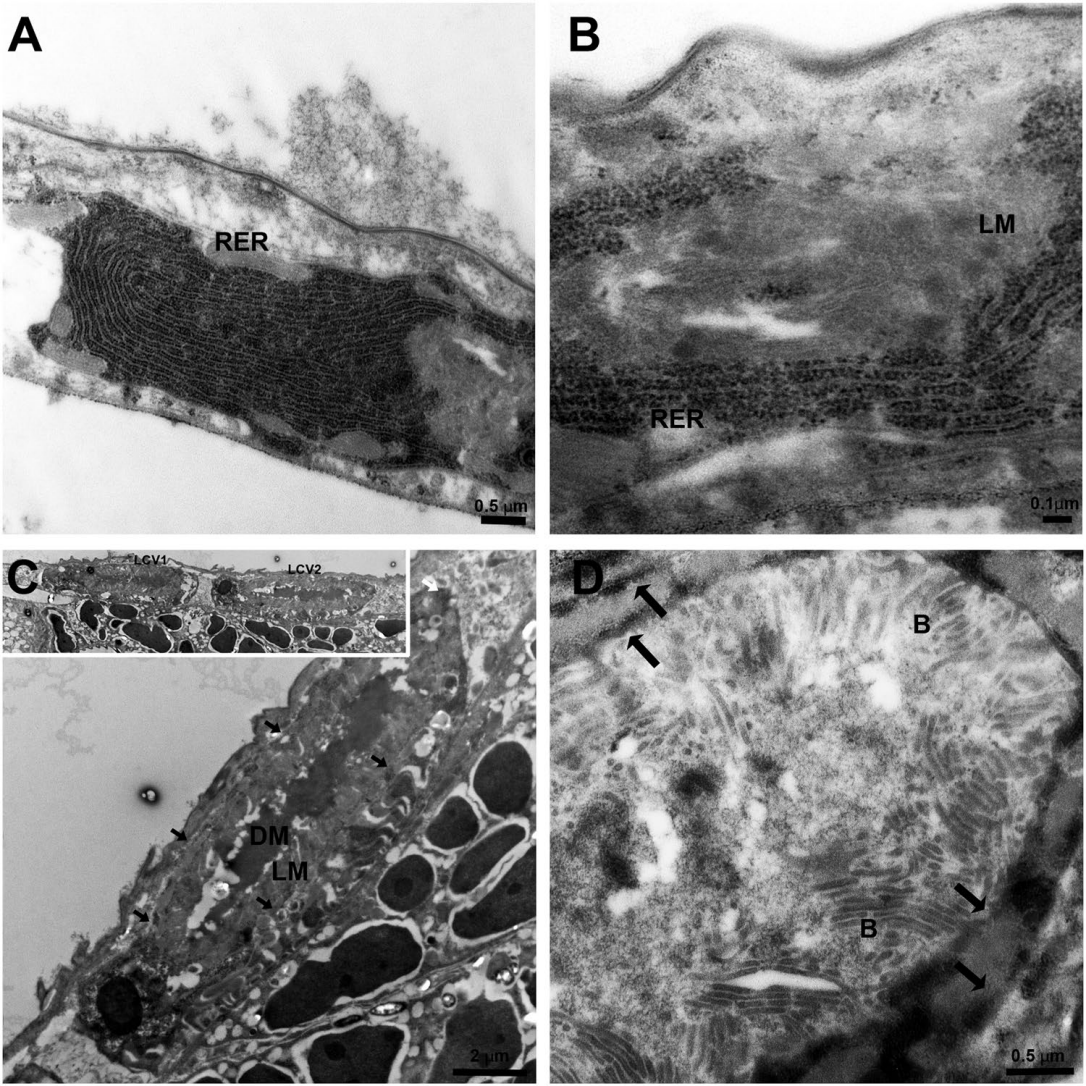
TEM of CLas and ER dynamics in *D*. *citri* infected gut cells. (**A**) Dense RER in healthy midgut epithelia cell. (**B**) Rearrangement of rough ER (RER) membranes around the light matter (LM) inside a midgut cell. (**C**) Liberibacter containing vacuoles (LCVs) showing the LM and the dark matter (DM) inside them. LCVs are located close to midgut basal lamina. Two adjacent LCVs are shown in the inset. LCV borders are indicated with arrows. (**D**) Zoom-in on one LCV showing mature CLas bacteria (**B**) in the LM, surrounded by the double membranous ER (arrows).

**Figure 3:**
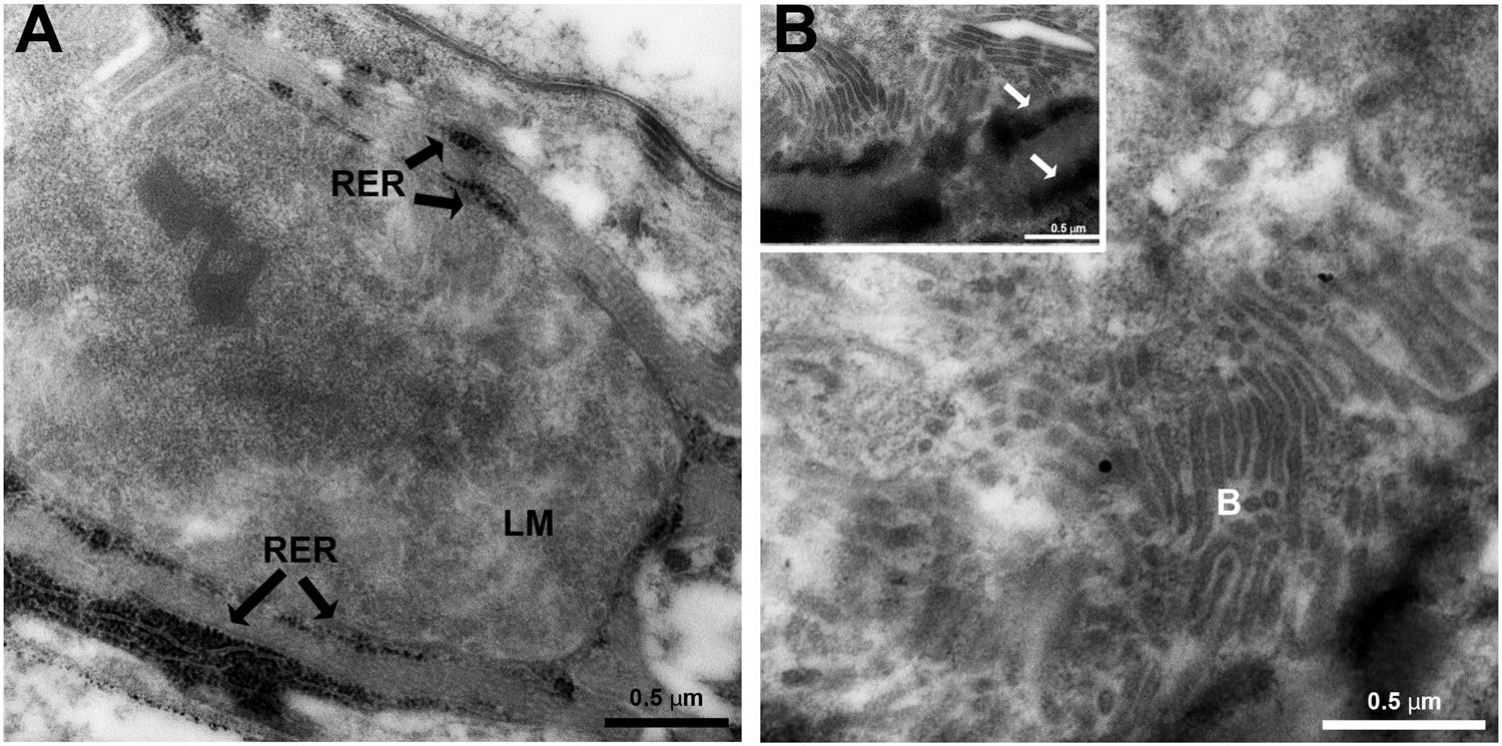
TEM images of LCVs in *D*. *citri* gut cells. (**A**) Structure of LCV with the light matter (LM) and the double-membrane layer of the ER (RER, arrows) that surrounds this body. (**B**) LCV showing the high density of bacteria inside the vacuole (**B**) and the dense double membrane layer of the ER (arrows in the inset).

**Figure 4:**
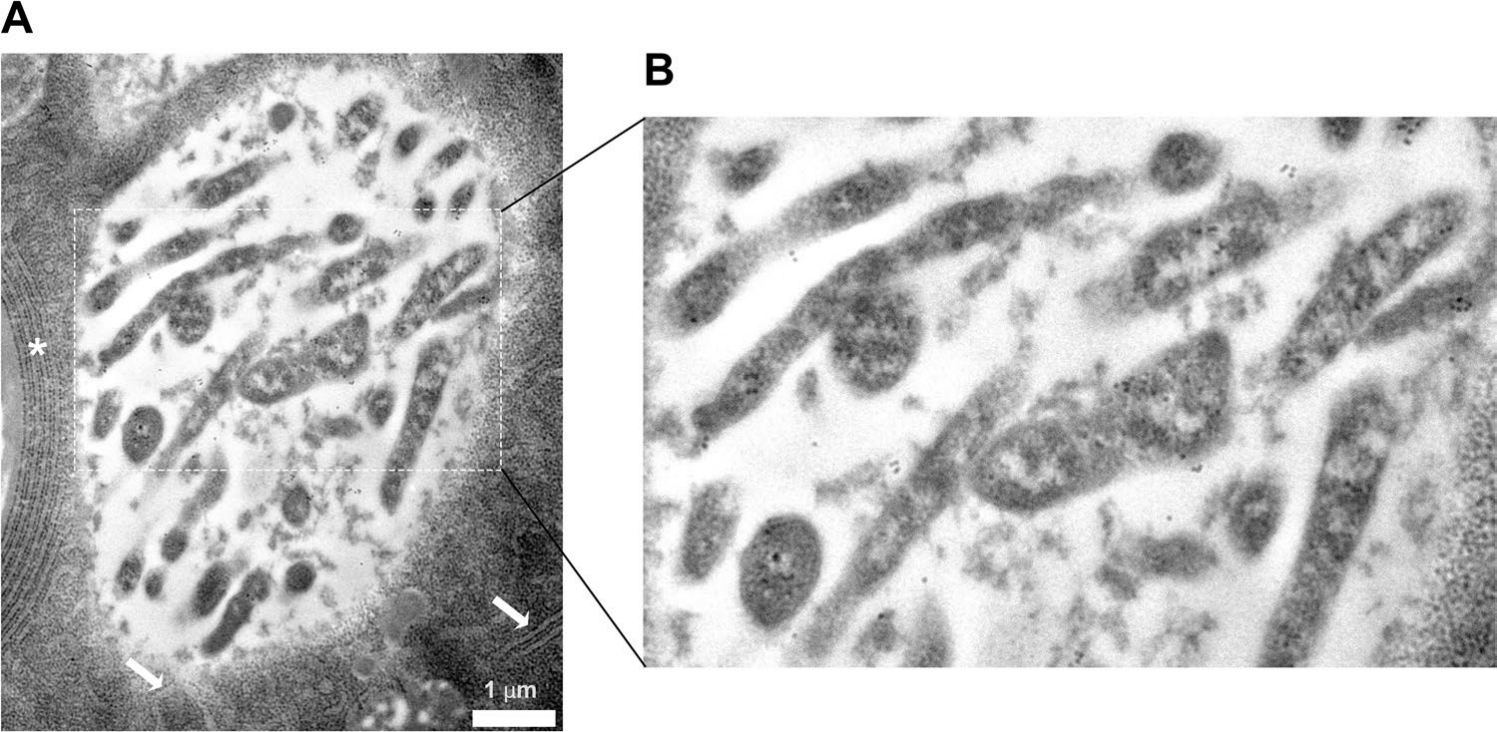
Immunogold labelling of CLas inside an LCV, using specific antibody against CLas outer membrane protein, OmpA. (**A**) Immunogold labelling of CLas inside vacuole, adjacent to the ER (white asterisk), and surrounded by ER membrane (white arrows). (**B**) An enlargement of the area marked by the dashed line in (**A**), showing gold labelling of CLas cells inside the LCV.

It has been shown that CLas moves into the midgut, and that high amounts of the bacterium are translocated from the midgut to the hemolymph during the transmission pathway. Thus, the TEM analysis was further extended to visualize the bacterium in ultrathin sections prepared from *D*. *citri* abdomens of insects that were continuously reared on CLas-infected plants. The results revealed a unique association between CLas and the ER (Figs 2 and 3). In epithelial cells, intensive ER could easily be visualized. Figure 2A shows typical Rough ER (RER) structure as normally seen in midgut epithelial cells. In CLas infected psyllids, we could detect the formation of vesicles filled with light matter (LM), and surrounded by two layers of RER (Figs 2B–D and 3A,B). In the center of these bodies, a dense matter (DM) was sometimes observed (Fig. 2C). Higher magnification images showed that these LM in these bodies was filled with bacterial cells that had the typical structure of CLas. The bacterial cells appeared elongated and rod-shaped or filamentous, as was previously described^5^ (Figs 2C,D, 3 and 4). When the bacteria were cross-sectioned, they looked round (Figs 3B and 4). In order to verify the presence of CLas inside the bodies, we performed immunogold labelling experiments with ultrathin TEM sections and an antibody against the outer membrane protein A (OmpA) protein of Liberibacter, which was previously shown to specifically label CLas^13,22,23^. Gold particles strongly associated with the bacteria inside the bodies, with some low level of unspecific binding, confirming the presence of CLas inside the ER-associated bodies (Fig. 4). These bodies were termed Liberibacter containing vacuoles (LCVs). Importantly, we could detect the LCVs in different developmental stages, with different sizes and different levels of CLas accumulation. While some LCVs appeared small and with a few bacterial cells (Fig. 2B), other LCVs were much bigger, and filled with bacteria (Fig. 2D). Our results strongly suggest that in *D*. *citri*, CLas replicated inside ER-associated LCVs.

**Figure 5:**
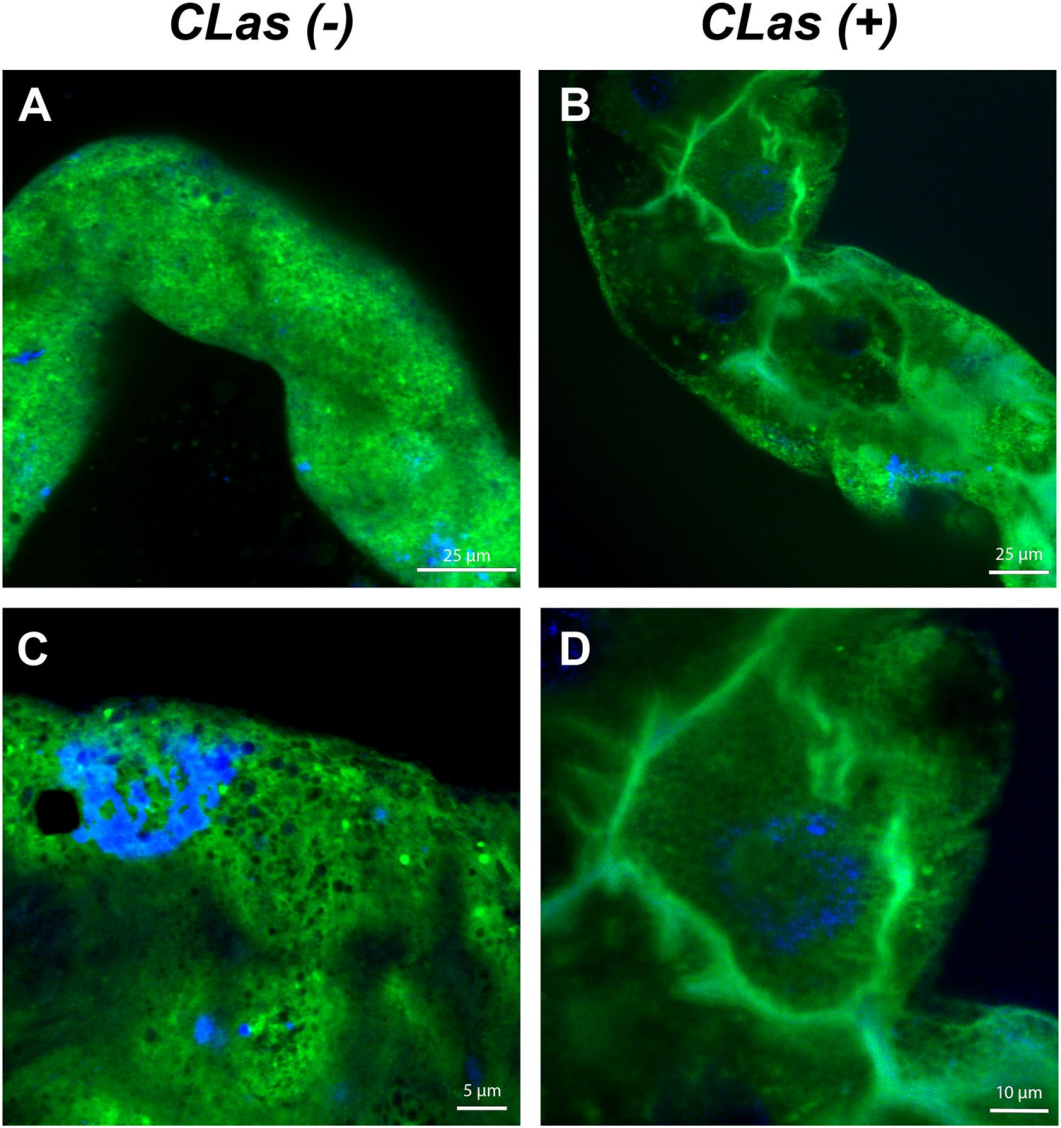
Rearrangement of the ER following infection with CLas. CLSM images of *D*. *citri* guts stained with ER tracker (green) from CLas-uninfected (**A**) and infected (**B**) *D*. *citri* adults. (**C**,**D**) show a higher magnification of a portion from the gut shown in (**A**,**B**) respectively. DAPI staining of the nuclei is shown in blue.

**Figure 6:**
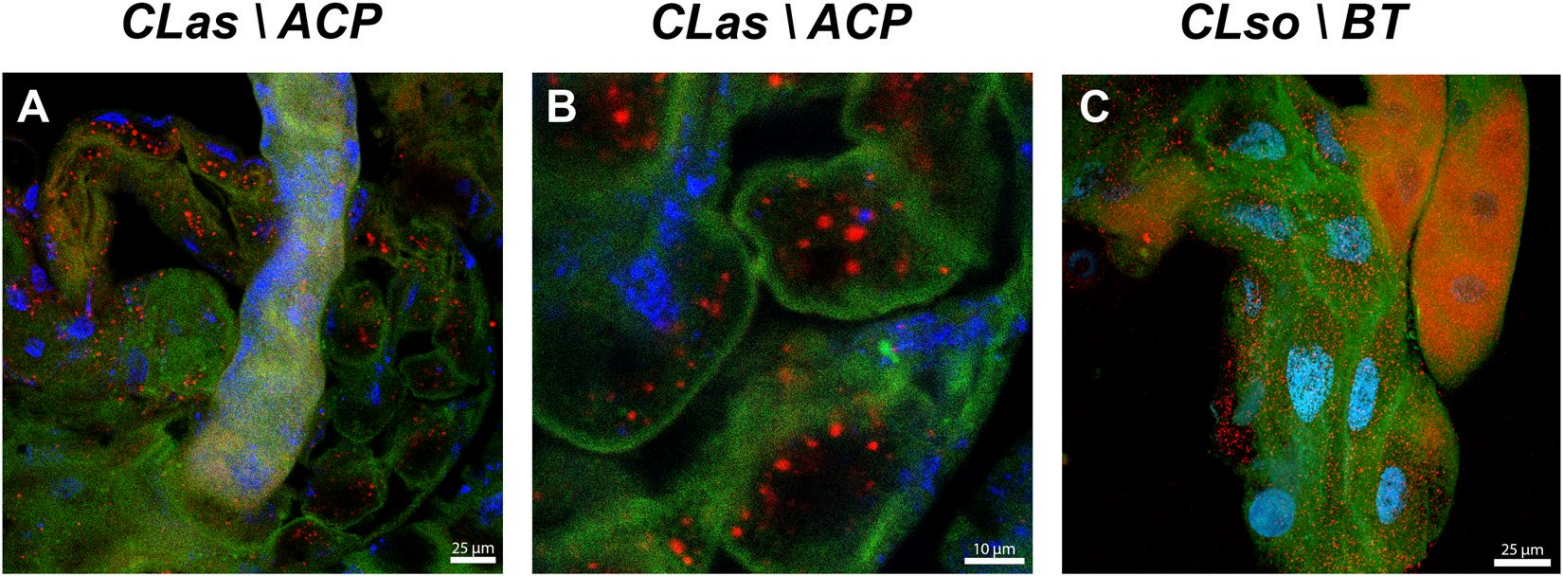
Formation of LCVs in CLas and CLso infected gut cells. CLSM images of *D*. *citri* guts stained with ER Tracker (green) together with CLas immunolocalization using a specific antibody against the outermembrane protein A (OmpA) of Liberibacter (red). Nuclei were stained with DAPI (blue). (**A**) CLas infected *D*. *citri* gut showing formation of ER intracellular bodies and immunolocalization of CLas inside ER bodies. (**B**) CLas immunolocalization inside ER associated LCVs and nuclei defregmantation. (**C**) ER staining in guts from CLso-infected *B*. *trigonica* adults show specific immulolocalization of CLso but no association with the ER or LCV formation.

To further confirm these results, we used the ER tracker fluorescent marker that stains the ER cellular compartments. In CLas- guts, ER was found throughout the cells and appeared normal (Fig. 5A). In higher magnification images, we could detect the common polygonal structure of the ER (Fig. 5C). In CLas+ guts, the ER showed a dramatic re-organization in the periphery of the cells, and in higher magnification images it was much harder to detect the polygonal structures, indicating that the ER rearranges to form intracellular bodies (Fig. 5B and D), and suggesting that the presence of CLas induced a re-formation of the ER. To confirm CLas association with the ER, we performed immunolocalization experiments using the antibody against the OmpA protein of Liberibacter together with ER staining. The results showed that CLas localizes inside vesicular structures that are formed from, or surrounded by, the ER membranes (Fig. 6A,B). Defragmentation of the nuclei was also sometimes observed in these cells (Fig. 6A,B). To test whether those phenotypes could also be observed in another psyllid-liberibacter system, we used the OmpA antibody and ER-tracker to detect CLso in midguts dissected from *B*. *trigonica* psyllids that were continuously reared on CLso-infected celery plants. Interestingly, CLso was not specifically associated with the ER, which appeared normal. CLso uniformly localized throughout the cell cytoplasm and did not associate with specific ER-associated intracellular structures (Fig. 6C). Our fluorescent microscopy results confirmed that in the *D*. *citri* guts, but not in *B*. *trigonica* guts, CLas is localized in vacuoles surrounded by ER membranes that we termed LCVs (Figs 5 and 6).

## Discussion

It was recently shown that CLas presence in midgut cells caused an apoptotic response that leads to defragmentation of cellular components, especially the nuclei^13^. This response is probably an antimicrobial one, and it was speculated that CLas might have evolved to exploit this immune response in order to exit the midgut cells on its transmission pathway. Even in the presence of this immune response, CLas was still shown to propagate inside the psyllid^12^ and this is especially significant when the bacteria is acquired at the nymphal stage^12^. This raises an important question that still needs to be answered: how is CLas able to propagate and avoid the gut cells immune response? In order to answer this question we took a microscopic approach and prepared ultrathin sections from abdomens of *D*. *citri* that acquired CLas, and then analyzed the gut cellular morphology under the TEM. We show here that in the adult midgut cells, CLas resides, and most likely replicates, inside membranous LCVs, and that these LCVs are actively recruiting the cellular ER endomembrane. We could only detect these bodies in the midgut cells, where they localized next to the basal lamina, ideally placed adjacent to their presumed exit sites to the hemolymph. The ER associated membrane bodies contain ribosomes, as was determined by the electron microscopy, and the ER that engulfs the bacterial vacuole has a double membrane structure. The double membrane nature of these bodies, and the presence of the ribosomes, suggest that they are generated from the rough ER, and that the bacteria does not enter the ER lumen, but rather recruits the ER which is wrapped around the bacteria vacuole.

Formation of ER-derived intracellular bodies, in order to form an independent isolated cellular niche that is not exposed to the host cellular immune machinery, is widespread phenomena that viruses use, and almost all viruses modify the host cellular membranes in order to form a viroplasm or virus replication body^24^. In plants, many viruses target specifically the ER membrane in order to replicate and move^25,26^, and similar effects were seen in insects that transmit viruses^27^. With bacteria, forming a replicative niche with the ER is less common, which is surprising because the ER can also provide bacteria with a safe and rich environment that is devoid of bactericidal, such as antimicrobial peptides or hydrolytic enzymes^15^. Nevertheless, there are few examples of bacteria that interact with the ER in order to subvert and escape the immune response of the host^28^. Two well-studied examples of such bacteria-host interactions are *Legionella pneumophila* and *Brucella spp*, the causal agents of the legionnaire’s disease and brucellosis, respectively. *Legionella* seizes early secretory vesicles that traffic between the ER and Golgi, which fuse with the Legionella containing vacuoles (LeCVs), and enable LeCVs to fuse with the ER membrane, and form a replication organelle that expands together with bacteria proliferation^29–32^. On the other hand, *Brucella* containing vacuoles (BCVs) first mature along the endocytic pathway, and later interact with ER exit sites, leading to biogenesis of ER-derived vacuoles through progressive exchange of endocytic membranes for ER-derived membranes^33,34^. As in LeCVs, the BCV support bacteria replication, which lead to a dramatic reorganization of the ER into replicative BCVs as bacteria proliferation continues. Our results shown here show that the LCVs we detected are also variable in size as previously observed for LeCVs. Presumably, the dark matter and the change in the matrix color of the LCVs as it appears in our images may hint on changes in the composition of this matrix which possibly provide the bacteria with the necessary environment for replication at early stages of the invasion. Later, the dark matter seems to disappear and the vacuole becomes filled with bacterial cells in preparation for exiting the cells (Figs 2 and 3).

Although *Legionella* and *Brucella* use two distinct mechanisms for the formation and development of their ER-associated replicative vacuoles, one element is identical for these bacteria- both pathogens express a type IV secretion system and use it to secrete effectors to the host cells, and this secretion system is required for the formation of the ER-associated vacuole^15,35,36^. CLas does not contain the type IV secretion system, but we speculate that it uses another system, such as the type I secretion system to secrete proteins into the host gut in order to form ER-containing vacuoles. CLas genome does not contain homologues for most of the known secreted effectors of *Legionella* and *Brucella*, such as DrrA, RalF and BspA-D, but does contain homologous for the LepB and VceC effectors, which were shown to be required for ER-associated vacuole formation in these pathogens. The identity of CLas effectors that are secreted in the gut cells of *D*. *citri* is still unknown.

We did not detect the LCVs in *B*. *trigonica* adults that were reared on CLso-infected plants. This could result from a low bacteria titer in these insects, which made the detection difficult. Interestingly however, CLso did not seem to induce similar apoptotic response as well (Fig. 1)^13^. This supports the assumption that LCV formation is indeed a bacteria survival mechanism, in which CLas transforms the phagosome into a non-bactericidal vacuole by interacting with the ER, and therefore LCV formation may protect CLas from the activation of apoptosis. We cannot however rule out the possibility that the role of ER-containing LCVs is to provide the bacteria with a nutrient-rich environment, rather than escaping the immune response. It is still unknown how exactly CLas enters and exits the gut cells to cross the gut barrier, but it was shown that one of the strongest differentially expressed pathways in infected *D*. *citri* is the endocytosis pathway^37^. An interesting possibility is that CLas employs endocytosis in order to enter the gut cells, and may take advantage of the apoptotic response in order to generate breaks in the cell membrane through which the bacteria can exit. Alternatively, it may use a similar pathway as was shown for CLso, where the bacteria was found in intact, stacked, vesicle-like structures on the outer basal laminar surface, suggesting that it uses exocytosis to arrive at the external midgut basal laminar surfaces^14^. Overall, our results suggest that the cellular processes and interaction that take place between CLas and *D*. *citri* in nymphs, CLas and *D*. *citri* in adults and CLso and *B*. *trigonica* may differ, and that a different strategy may be employed by both the insect and the bacteria in each type of interaction.

In summary, in this study we were able to detect CLas inside the gut of *D*. *citri*, and identified a new level of interaction between the two organisms. Our study highlights the close association between CLas and *D*. *citri*, and supports the view of CLas as an insect pathogen^38^. The formation of ER-associated LCVs in the psyllid strongly suggests that CLas secretes effector proteins while inside the psyllid gut, that can manipulate the cellular responses for its own needs. Identifying the cellular targets that are manipulated in order to invade the gut cells and to generate the LCVs can provide novel targets for RNAi treatments^39^ to inhibit or even abolish CLas establishment inside the insect, and prevent the transmission.

## Electronic supplementary material

Supplementary Information 
