# Supplementary material for: ‘Candidatus Liberibacter asiaticus’ Accumulates inside Endoplasmic Reticulum Associated Vacuoles in the Gut Cells of Diaphorina citri
Source: Sci Rep. 2017 Dec 5;7:16945. doi: 10.1038/s41598-017-16095-w (PMC5717136; doi:10.1038/s41598-017-16095-w)

Murad Ghanim, Diann Achor, Saptarshi Ghosh, Svetlana Kontsedalov, Galina Lebedev, Amit Levy: ***'Candidatus* Liberibacter asiaticus' Accumulate Inside Endoplasmic Reticulum Associated Vacuoles in the Gut Cells of *Diaphorina citri*.**.

Supplementary Material:

Figure S1. TEM of nuclei and chromatin architecture in CLas- and CLas+ *D. citri* midguts. (A) Normal nucleus from CLas-uninfected gut, showing the defined boundaries of the nucleus, the chromatin inside and the nucleolus. (B) and (C) show abnormal nuclear structures in *D. citri* infected with CLas. ER- endoplasmic reticulum, N- nucleus, Nu-nucleolus, NM- nuclear membrane.

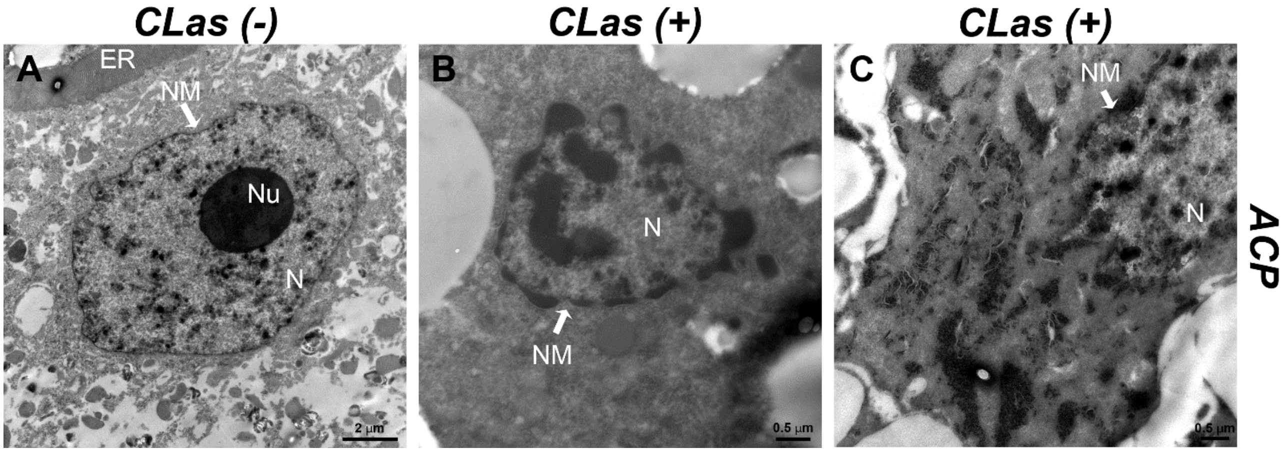

Supplement: Supplementary file 1 — Supplementary Information [file 41598_2017_16095_MOESM1_ESM.pdf]
